# Effect of the Ratio of Magnetite Particle Size to Microwave Penetration Depth on Reduction Reaction Behaviour by H_2_

**DOI:** 10.1038/s41598-018-33460-5

**Published:** 2018-10-09

**Authors:** Ahmadreza Amini, Ko-ichiro Ohno, Takayuki Maeda, Kazuya Kunitomo

**Affiliations:** 1 Department of Materials Process Engineering, Graduate School of Engineering, Kyushu University, Kyushu, Japan; 2 Department of Materials Science and Engineering, Faculty of Engineering, Kyushu University, Kyushu, Japan

## Abstract

In this study, we investigated reduction of magnetite by H_2_ during microwave irradiation. This process combines the advantages of microwave irradiation and using H_2_ as a reducing agent to mitigate CO_2_ emissions during the ironmaking process. Weight change measurements showed that a reduction of 75% was achieved after treatment under H_2_ for 60 min. For better understanding of the effective parameters in microwave chemistry, scanning electron microscopy, combined with energy-dispersive X-ray spectroscopy (SEM-EDX), was performed, which demonstrated a greater reduction of large particles (>40 μm) than small particles. This behaviour could be attributed to the higher microwave absorption capability of large particles with a higher ratio of particle size to penetration depth (*d/δ*). Small particles behave as transparent material and are heated via conduction and/or convection; thus, there is no contribution from the catalytic effect of microwaves to the reduction reaction. Moreover, the reduction of Fe_3_O_4_ to Fe_0.94_O, followed by transformation to Fe, seems to proceed from the surface toward the centre of the particle despite the volumetric microwave heating. This could be due to the higher gas accessibility of iron oxide on the particle surface than in the particle centre.

## Introduction

Microwave irradiation has the potential to decrease the amount of carbonaceous materials required for eco-friendly chemistry owing to its specific characteristics such as non-thermal effect (catalytic effect)^1–4^, volumetric heating, rapid and selective heating^3^ and high-efficiency heating^5^. For example, the theoretical carbon consumption in conventional heating such as blast furnace is ~380 kg per t of pig iron production, where ~230 kg is used for iron oxide reduction, and the remainder (150 kg) is used for heating and preparing the reaction energy^6^. The energy required to break the iron–oxygen bonds is 10^5^ times higher than the energy that can be provided by microwave photons, i.e. 10^−5^ eV. In an antibonding state of an unpaired spin, the microwave can vibrate the state causing enhancement in deoxidation^4^. Moreover, Hayashi *et al*.^7^ reported that the flow of conductive heat from the surface to the centre is the rate controlling step during the reduction of powdery iron oxides. They employed the volumetric heating characteristics of microwave heating to overcome this problem. Kashimura *et al*.^4^ took advantage of the high efficiency of microwave heating to produce iron from an Fe_3_O_4_ and graphite mixture. They found that the combined effect of thermal energy and magnetic field of microwaves improves deoxidation during carbothermic reactions. Chun *et al*.^8^ showed that using microwave heating for iron oxide reduction by carbon leads to a porous structure due to the rapid heating of both iron oxide and carbon by microwave irradiation. Stir *et al*.^9^ studied the reduction behaviour of magnetite by carbon black at the E-field maxima position of a microwave generator, and found that formation of primary and secondary wüstite represents intermediate steps in the reduction of Fe_3_O_4_ to Fe.

Previously, a mixture of iron oxide and carbonaceous material has typically been used to study iron production during microwave heating due to the susceptibility of carbon to microwaves. The amount of carbonaceous material required in this method is less than that of the conventional ironmaking process^6,10^. Demand for reduced CO_2_ emissions during the ironmaking process has prompted research into a cleaner and more efficient method of iron production. One possibility is to employ H_2_, instead of carbonaceous materials, as the reducing agent^11,12^ to mitigate CO_2_ emissions. The reduction rate of iron oxide in H_2_ is much higher than that in CO^12–16^ and Murakami *et al*.^17^ showed that the interdiffusion coefficient of H_2_/H_2_O gas in iron ore sintered particles at 500 °C is three times higher than that of CO/CO_2_. Considering these advantages of using H_2_ as the reducing agent, several previous studies have investigated iron oxide reduction by H_2_. Fruehan *et al*.^18^ studied the final stage of iron ore reduction by H_2_ and showed that formation of an iron layer around large grains causes a change in the reaction mechanism, from a gas reaction to solid state oxygen diffusion. Pineau *et al*.^14^ demonstrated that Fe_3_O_4_ is reduced directly by H_2_ to Fe at temperatures lower than 420 °C whereas, in the temperature range of 450–570 °C, magnetite, wüstite, and iron can co-exist due to a decrease in the apparent activation energy of the reduction reaction. This was explained by decreased defects in the crystalline structure of magnetite, which can be annealed at ~420 °C. Other researchers^16,19–21^ reported an increase in the reduction of magnetite powder by H_2_ in the presence of a strong external magnetic field. Kim *et al*.^16^ and Raw *et al*.^21^ theorized that the reduced iron particles became rearranged in the magnetic field, preventing their agglomeration and increasing the access of the reducing reagent H_2_ to the unreacted magnetite powders.

Therefore, eco-friendly ironmaking would involve non-carbonaceous materials, such as H_2_, in the reduction reaction via microwave irradiation. In this case, only magnetite is employed as the initial microwave absorber and its transformation to wüstite or metallic iron via the reduction reaction could have a significant effect on microwave absorption. Furthermore, the non-thermal effect of microwave irradiation on the rate of chemical reactions, referred to as the catalytic effect of microwaves, has attracted the attention of researchers on microwave irradiation energy in terms of speeding up chemical reactions^1–4^. However, such an effect cannot be considered when target particles cannot absorb microwaves and behave like transparent materials. Therefore, to improve our understanding of microwave chemistry, effective parameters of microwave absorption and the subsequent reaction should be identified, and the extent of each effect needs to be investigated. For instance, it has been reported that the ratio of the particle size, *d*, to the microwave penetration depth, *δ*, is a critical factor for optimum microwave absorption by conductive materials^22,23^. The penetration depth, *δ* (*m*), represents the distance from the surface to a point inside the material where the power of the exposed electromagnetic waves decreases to 1/e (36.8%) of the surface value^23,24^, which can be calculated using Equation (1) ^24,25^:

1 $$\delta ={[\frac{{\omega }^{2}\varepsilon ^{\prime} \mu ^{\prime} }{2}(\sqrt{1+\frac{{\sigma }^{2}}{{\omega }^{2}{\varepsilon ^{\prime} }^{2}}}-1)]}^{-\frac{1}{2}}$$

where *σ* (S/m) is the electrical conductivity, *ω* (rad/m) is the angular frequency of the microwave, and *ε′* (dimensionless) and *μ′* (dimensionless) are the real parts of permittivity and permeability, respectively. At a very low *d/δ* ratio, the particle acts as a transparent material and cannot couple with the microwaves. By contrast, at a very high *d/δ* ratio, most of the microwaves will be reflected and the sample cannot heat well. For example, a *d/δ* value of ~2.4 has been suggested for optimum microwave absorption in non-magnetic metal particles^22,23^. In our previous study^25^, the interaction between microwaves and magnetite particles was investigated in neutral atmosphere (N_2_) to clarify the effect of particle size and apparent density of a magnetite sample on microwave absorption.

In this study, the potential for magnetite reduction by H_2_ with microwave heating is investigated using a multi-mode microwave generator operated at an output power of 1050 W. Moreover, the effect of new-phase formation during Fe_3_O_4_ reduction on microwave heating is determined, and the effect of the *d/δ* ratio on the reduction reaction is elucidated.

## Methods

### Materials

The purpose of this study is to investigate the effect of the ratio of the magnetite particle size to microwave penetration depth on the reduction reaction by H_2_. Thus, a magnetite sample with known grain size distribution is required. At the laboratory scale, larger particles with known range of grain size can be prepared by heat treatment of finer particles followed by crushing, grinding, and sieving. Therefore, to prepare a magnetite sample with a grain size of less than 45 μm, a procedure similar to that used in our previous study^25^ was employed, wherein the reagent magnetite powder (particle size ≈1 μm, purity 99%, Mitsuwa’s Pure Chemicals, JAPAN) was pressed into a tablet shape (30 mm in diameter, 20 mm in height), followed by heating in flowing Ar at 1350 °C for 60 min in an electric resistance furnace. Ar was used to provide an inert atmosphere to avoid variation in the oxidation state of magnetite. The heat-treated tablets were crushed and ground to a grain size less than 45 μm. Then, to prepare the briquette samples, ~0.02 g of a 5 mass% aqueous solution of polyvinyl alcohol (Tokyo Chemical Industry CO., LTD., JAPAN) was added as a binder to 3 g of the crushed magnetite powder. Then, a cylindrical briquette sample (15 mm in diameter, 5 mm in height, 37% porosity) was formed by a cold press and was dried at 120 °C for 10 h. Pure H_2_ gas was used as the reducing agent after removing its humidity by passing it through a silica gel column.

### Microwave irradiation

**Figure 1:**
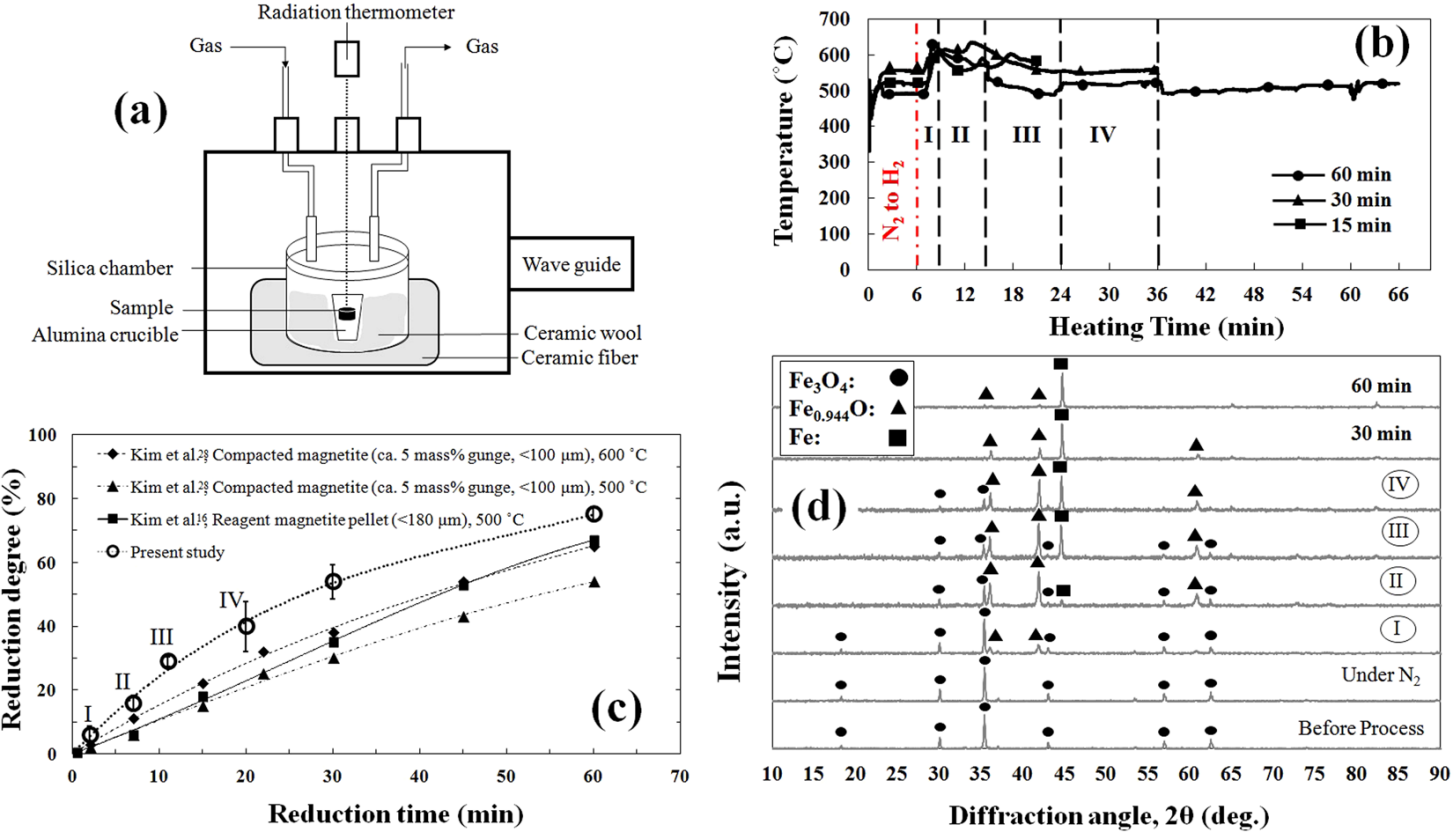
(**a**) Schematic setup for microwave irradiation. (**b**) Typical temperature profiles of microwave-heated magnetite samples during reduction by H_2_ for 15, 30, and 60 min. The dash-dotted line shows the time the atmosphere was changed from N_2_ to H_2_. Dashed lines delineate the four heating stages (I, II, III, and IV) determined during the experiment. (**c**) Reduction degree of the magnetite samples reduced by pure H_2_ in the present study (microwave irradiation) and reported by other researchers (conventional heating). Error bars represent the standard deviation of the average reduction degree for a certain treatment duration. (**d**) XRD patterns of magnetite samples before, during, and after reduction by H_2_ during microwave irradiation.

Prepared briquette samples were subjected to microwave irradiation using a multi-mode microwave generator with a maximum output power of 1.5 kW at 2.45 GHz under N_2_ (1 NL/min) atmosphere, in which both the perpendicular magnetic (H) and electric (E) fields contribute to heating (Fig. 1a). The atmosphere was changed to H_2_ (1 NL/min) after 6 min, which was designated as time zero for reduction degree calculations. A constant power of 1050 W was manually set until the end of heating in all experiments using three tuning stubs.

The Fe_3_O_4_ sample was contained in an alumina crucible (41 mm outer diameter (OD), 36.5 mm inner diameter (ID), and 49 mm height) surrounded by ceramic wool (Al_2_O_3_: 35%, SiO_2_: 50%, and ZrO_2_: 15%, Isolite Insulating Products CO., LTD., Japan). The crucible was then placed in a silica chamber (95 mm OD, 85 mm ID, 96 mm height). Ceramic wool, which cannot absorb microwaves, served as a thermal insulator to prevent heat loss during heating and to protect the silica chamber. The sample temperature was measured using an infrared thermometer capable of measuring a temperature range from 330–1500 °C. The silica chamber was purged by pure N_2_ gas before the experiment. Each experiment was conducted for a certain time, and then microwave irradiation was stopped and the atmosphere was changed to N_2_ for the cooling step. The cooled sample was subjected to weight change measurement, XRD, SEM-EDX, and optical microscopy.

### Sample analysis

To identify the phase transformations during heat treatment, briquette samples were subjected to phase analysis using X-ray diffraction (XRD, Cu-*Kα*; *λ* = 1.54 Å; scan speed, 51.9°/min; power, 3 kW; RIGAKU Smartlab, ZOTK, JAPAN) before and after the reduction reaction. For this purpose, a portion of the treated sample was ground in a ceramic mortar with a pestle to a fine powder (93 mass% under 32 μm, 5 mass% 32–45 μm, and 2 mass% 45–75 μm). Further, the microstructure of the remaining parts of reduced samples was analysed using an optical microscope (BX 50, OLYMPUS, JAPAN) and SEM-EDX (SU 3500, HITACHI, JAPAN).

## Results and Discussion

Figure 1b shows typical temperature profiles of microwave-heated Fe_3_O_4_ samples during reduction by H_2_ for 15, 30, and 60 min. A similar temperature profile was observed for all samples, confirming the reproducibility of the method. An unsteady temperature profile after changing the atmosphere to H_2_ demonstrated some variation in the microwave absorption of the samples at different stages of reduction. Such unsteady heating behaviour had also been observed in previous studies^4,26–28^. However, in those studies, a gradual increase in temperature during microwave heating was reported, which would be due to the presence of carbonaceous materials as a microwave susceptor in their samples. In this study, each significant change in the heating behaviour of the sample was considered a distinct stage (stages I, II, III, and IV) and was used to study the effect of new phases formed via the reduction reaction on the microwave absorption capability of the sample.

The reduction degree, %R (dimensionless), was calculated according to Equation (2):

2 $$ \% R=\frac{{W}_{i}-{W}_{t}}{{W}_{O}\times {W}_{i}}$$

where *W*_*i*_ (g) is the initial weight of the sample, *W*_*t*_ (g) is the weight of the sample after treatment for *t* min, and *W*_*O*_ (dimensionless) is the stoichiometric weight ratio of oxygen in magnetite, which is 0.2766. Figure 1c shows the reduction degree of magnetite samples after different treatment durations in the present report and that achieved by other researchers^16,29^. To validate the results, each experiment was conducted at least twice. The average reduction degree was used as the reduction degree for a certain treatment duration in Fig. 1c, in which the error bars represent the standard deviations. Evaluations of the reduction degree based on weight change measurements (Fig. 1c) indicate that the reduction reaction began soon after changing the atmosphere to H_2_. Therefore, as mentioned above, phase transformation during the reduction reaction was responsible for the unsteady heating behaviour of the sample. The XRD patterns of the samples before, during, and after reduction by H_2_ during microwave irradiation are illustrated in Fig. 1d for different treatment durations.

According to the XRD pattern of the sample treated in the first 6 min under N_2_, no phase transformation was detected, demonstrating heating of the sample without any reduction.

In stage I, Fe_0.94_O was detected in the XRD pattern, confirming the initial stage of the reduction reaction. An increase in the sample temperature after changing the atmosphere to H_2_ showed some variation in microwave coupling with the sample owing to wüstite formation during this stage. For improved coupling of materials with irradiated microwaves, microwave power absorption, *P* (W/m^3^), and microwave penetration depth, *δ* (m), could be considered the most effective parameters.

The *P* (W/m^3^) value of a material represents its ability to convert microwave power into thermal energy, which can be evaluated using Equation (3) ^24,25,30^:

3 $$P=\frac{1}{2}\sigma {|E|}^{2}+\pi f{\varepsilon }_{0}{\varepsilon }_{r}^{^{\prime\prime} }{|E|}^{2}+\pi f{\mu }_{0}{\mu }_{r}^{^{\prime\prime} }{|H|}^{2}$$

where *f* (Hz) is the microwave frequency, *E* (V/m) is the electric field amplitude, *H* (A/m) is the magnetic field amplitude, *ε*_0_ (F/m) and *μ*_0_ (N/A^2^) are the permittivity and permeability of the vacuum, respectively, and ${\varepsilon }_{r}^{^{\prime\prime} }$ (dimensionless) and ${\mu }_{r}^{^{\prime\prime} }$ (dimensionless) are the imaginary parts of permittivity and permeability, respectively. According to Equation (3), three different mechanisms, i.e. Joule loss (first term), dielectric loss (second term), and magnetic loss (third term), contribute to microwave heating.

The Joule loss in wüstite is reportedly higher than that in magnetite owing to the higher electrical conductivity of wüstite (9.1 S/cm) than that of magnetite (1.0 × 10^−3^ S/cm)^4,31^. Moreover, Hotta *et al*.^32^ showed that not only the electrical conductivity but also the permittivity (real part) of wüstite is higher than that of magnetite. This leads to greater penetration depth in wüstite than in magnetite, as also reported by Peng *et al*.^33^. Therefore, higher microwave absorption is expected by wüstite, causing an increase in temperature after wüstite formation during stage I. This result is in good agreement with that of Ishizaki *et al*.^27^, where wüstite formation is considered responsible for an increase in temperature during microwave heating.

Metallic iron formed in stage II (Fig. 1d), which would be responsible for a decrease in sample temperature. It is well known that the penetration depth of metals like copper (*δ*_*Copper*_ = 1.3 μm) is substantially less than that of metal oxides such as magnetite (*δ*_*Magnetite*_ = 80 μm)^24^ because of the higher electrical conductivity of the former materials^23^. The formation of metallic iron in stage II resulted in less absorption of microwaves^27^ due to a shorter penetration depth than either wüstite or magnetite. Lower microwave absorption then led to a decrease in sample temperature during this stage.

In stage III, the intensity of the Fe peak increased, demonstrating greater reduction, which was also confirmed by weight change measurements (Fig. 1c). In stage IV, the sample temperature reached a steady state due to heat loss via conduction from the reaction chamber, equal to the heat generated in the sample via microwave irradiation. This phenomenon was also observed by Hayashi *et al*.^24^, who reported a constant temperature during microwave heating of magnetite and theorized that the rate of microwave absorption is equal to the rate of thermal energy dissipation. Moreover, comparing the reduction degree obtained in the present study with that reported by other researchers^16,29^ demonstrates that a greater reduction is achievable by employing microwave irradiation during magnetite reduction by pure H_2_ than by using conventional heating methods. Such behaviour is attributed to both thermal and non-thermal (catalytic) effects of microwave irradiation when speeding up chemical reactions, as demonstrated by other researchers^1–4^.

### Microstructural observations

**Figure 2:**
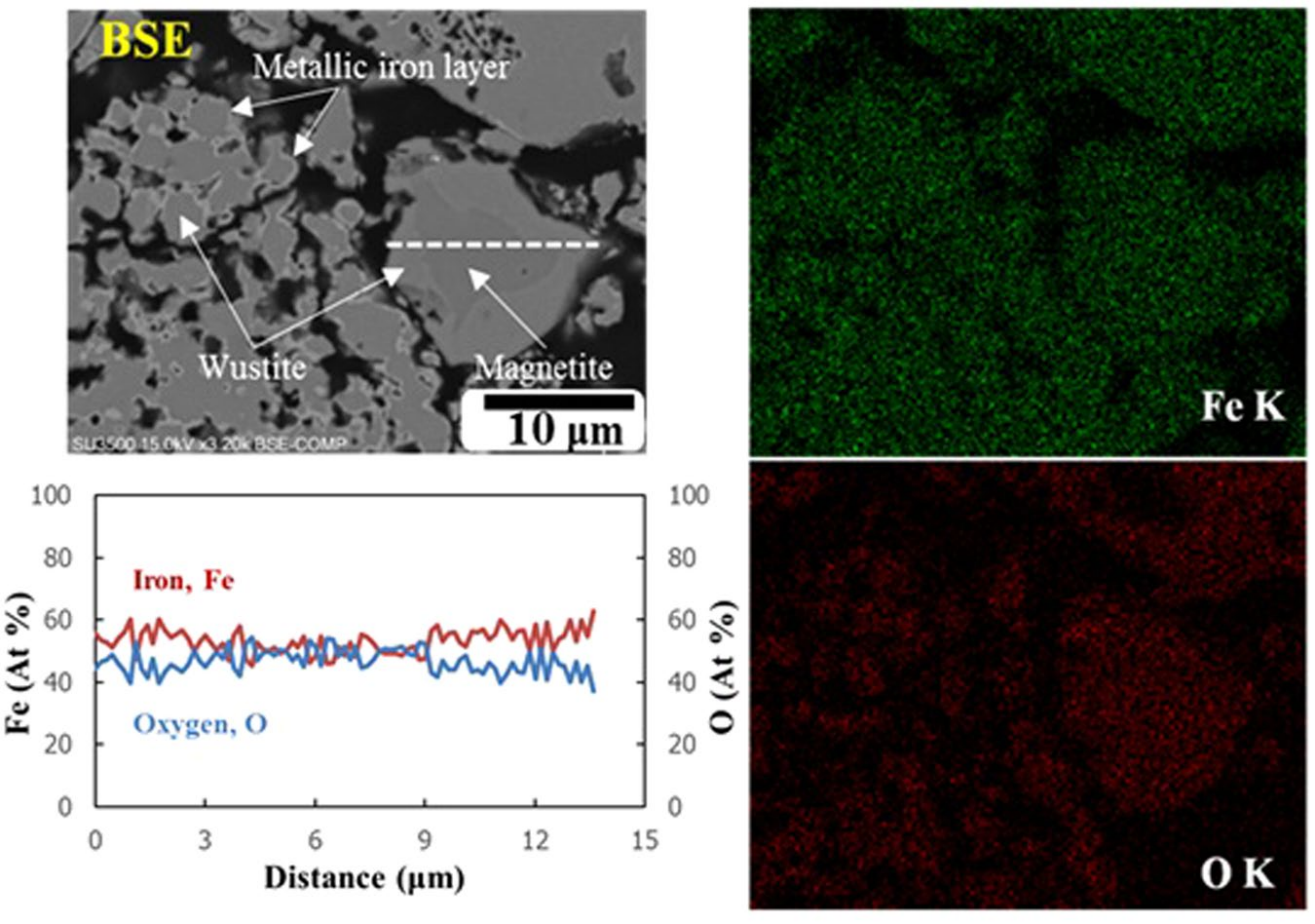
SEM-EDX map and line analysis of particles in the Fe_3_O_4_ sample reduced by H_2_ during 30 min microwave heating.

To identify the reduction mechanism of different particles, microscope observations were conducted. The SEM-EDX map and line analysis of particles in the Fe_3_O_4_ sample reduced by H_2_ during 30 min of microwave heating are shown in Fig. 2.

**Figure 3:**
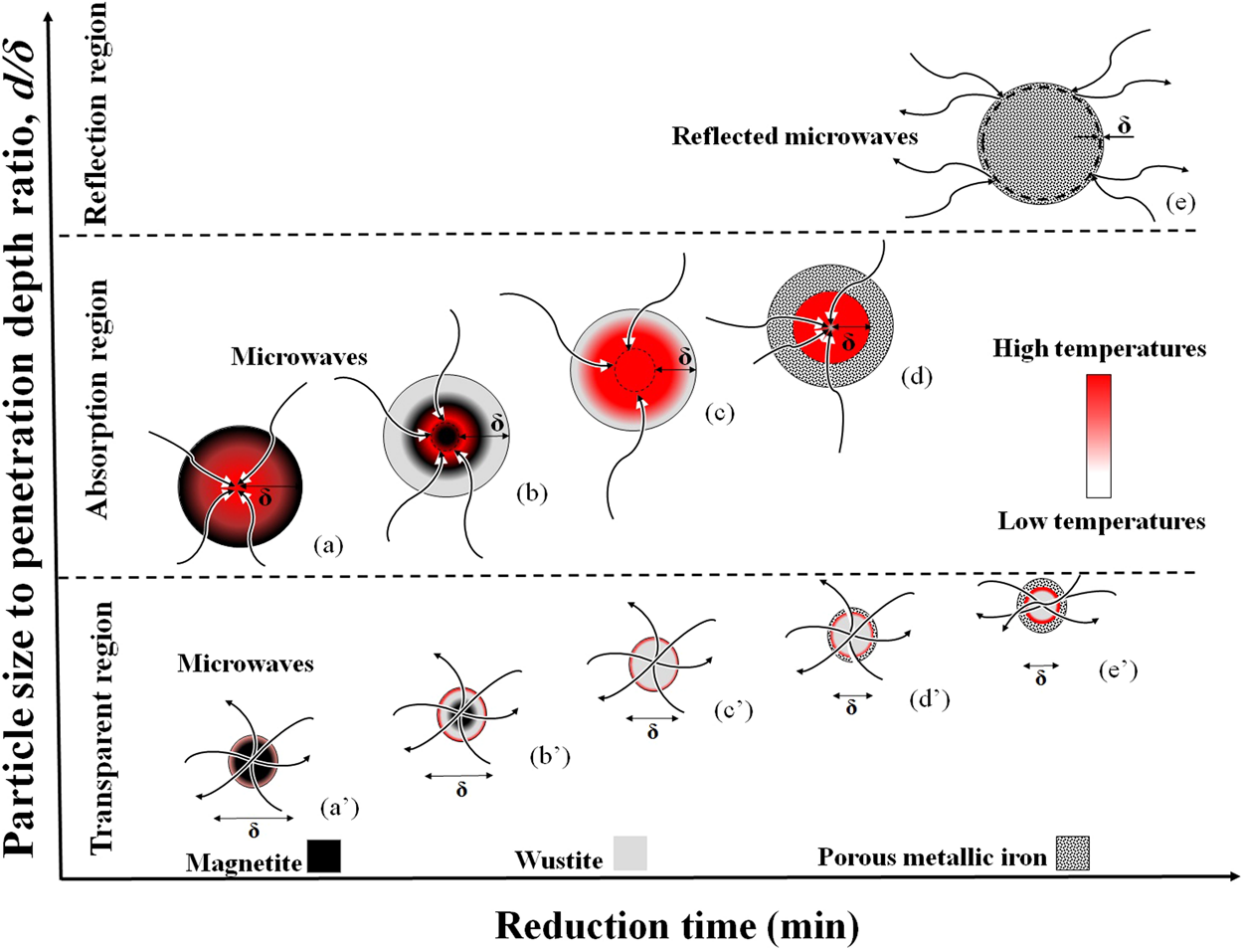
Mechanism of magnetite reduction by H_2_ during microwave heating. Dark grey: magnetite (Fe_3_O_4_). Bright grey: wüstite (Fe_0.94_O). Speckled pattern: porous metallic iron (Fe).

Very small particles (2–5 μm) were completely reduced to wüstite and the reduction reaction progressed via formation of a thin metallic iron layer on the particle surface. In small particles (~15 μm), the complete reduction of magnetite to wüstite required longer treatment time and no metallic iron was observed in these particles. These observations demonstrate that metallic iron can only form after the complete reduction of Fe_3_O_4_ to Fe_0.94_O. This result is in good agreement with that of Pineau *et al*.^14^, who reported that Fe_3_O_4_ was completely reduced to wüstite before reduction to Fe at temperatures above 570 °C. Moreover, reduction in small particles proceeded from the particle surface toward the particle centre in spite of the microwave irradiation. As mentioned above, the optimum *d/δ* ratio is known as an essential factor for optimum microwave coupling of conductive materials^22,23^. A penetration depth of ~80 μm was calculated by Hayashi *et al*.^24^ for magnetite powders at room temperature at a frequency of 2.45 GHz, where *ε′* is ~40, *μ′* is ~1.7, and *σ* is 1.0 × 10^2^ (S/cm), according to equation (1). The penetration depth for a compacted magnetite sample, as employed in this study, is less than 80 μm due to an increase in both permittivity and electrical conductivity with increasing relative density, as reported by other studies^23,24,32,34–36^. Therefore, the mechanism of magnetite reduction by H_2_ during microwave heating is a function of the *d/δ* ratio, as illustrated in Fig. 3.

**Figure 4:**
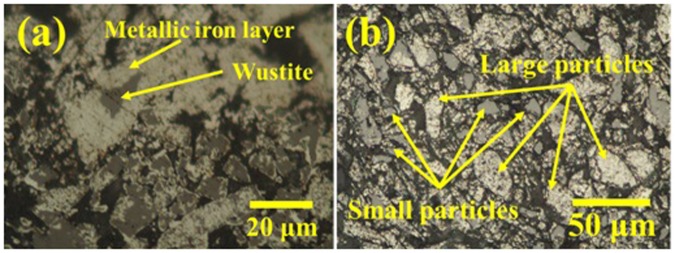
Optical microscope images of large particles in the Fe_3_O_4_ sample after treatment in H_2_ with microwave irradiation for (**a**) 30 min, partially reduced particle, and (**b**) 60 min, fully reduced large particle.

In particles larger than ~40 μm, magnetite was completely reduced to wüstite and then transformed to porous metallic iron. In these large particles, better microwave absorption is expected due to a higher *d/δ* ratio than that of small particles (Fig. 3a,a’). However, because of higher gas accessibility on the particle surface than the particle centre, as also reported by other studies^16^, it is expected that, even in large particles, the reduction of magnetite to wüstite (Fig. 3b,c) and of wüstite to porous metallic iron (Fig. 3d,e) proceeds from the surface toward the centre despite their good microwave absorption. This reduction direction has also been observed in previous studies^4,16,26,27,37^. This mechanism was confirmed by optical microscopy observations of a partially reduced sample after 30-min treatment under H_2_ (Fig. 4a), where an iron layer was observed around the wüstite.

Small particles with a very low *d/δ* ratio behaved as a transparent material during microwave irradiation in this study (Fig. 3a’) and were heated by transferring the heat from well-heated large particles via conduction and/or convection, instead of by microwave absorption. In addition, the catalytic effect of microwaves (non-thermal effect)^3^ on iron oxide reduction^4^ should be ignored in these small particles. In this case, a wüstite layer forms on the surface of the magnetite because of the reduction direction of magnetite to wüstite (Fig. 3b’,c’) and that of wüstite to porous metallic iron (Fig. 3d’,e’). This mechanism was confirmed by SEM observations (Fig. 2) of a wüstite layer on the surface of the magnetite in particles of ~15 μm, as well as a thin metallic iron layer detected on the surface of small particles.

On the other hand, Stir *et al*.^9^ demonstrated that the reduction kinetics of Fe_3_O_4_ to wüstite are controlled by the phase boundary while those of wüstite to Fe are affected by cation vacancies and structural defects. Thus, microwave irradiation would improve the reduction of wüstite particles, which are cubic crystal with Fe^+2^ defects^8^. Therefore, a higher temperature of the large particles (thermal effect) and a larger catalytic effect of microwaves (non-thermal effect)^3^ on iron oxide reduction^4^ would cause greater reduction in large particles than in small particles. Optical microscope observations of a reduced sample after 60 min of treatment under H_2_ (Fig. 4b) confirmed that the reduction progressed more in large particles than in small particles.

As mentioned in Section 3, the formation of metallic iron causes a significant decrease in penetration depth^24^ owing to the higher electrical conductivity of metals than oxides^23^. Therefore, the *d/δ* ratio increases noticeably, causing reflection of microwaves, as shown in Fig. 3e. Thus, microwave absorption should decrease with formation of metallic iron, as reported by other researchers^27^. This mechanism is confirmed by the decrease in sample temperature in stage II shown in Fig. 1b, where the XRD patterns demonstrate the formation of metallic iron (Fig. 1d).

## Conclusions

The possibility of magnetite reduction by H_2_ during microwave heating was investigated for the first time using a multi-mode microwave generator operated at an output power of 1050 W, to combine the advantages of microwave heating and using H_2_ as a reducing agent to mitigate CO_2_ emissions during the ironmaking process. In addition, the microstructure of the reduced samples was analysed to clarify the effect of the ratio of magnetite particle size to microwave penetration depth (*d/δ*) on the reduction reaction behaviour by H_2_. The results are summarized as follows:

1. The Joule loss in wüstite is higher than that in magnetite due to the higher electrical conductivity of wüstite (9.1 S/cm) than that of magnetite (1.0 × 10^−3^ S/cm). Therefore, wüstite formation via reduction reaction causes an increase in microwave absorption of the sample, leading to an increase in temperature.
2. Large particles, which have a high *d/δ* ratio, could couple with the microwaves, producing higher temperature and higher catalytic effect of microwaves on the reduction reaction progress in large particles. However, small particles, which have very low *d/δ* ratio, behave as a transparent material, and are heated via convection and/or conduction in the absence of either the thermal or non-thermal effects of microwave irradiation.
3. During microwave heating in a H_2_ atmosphere, the reduction of Fe_3_O_4_ to Fe_0.94_O and that of Fe_0.94_O to Fe proceeds from the particle surface toward the particle centre due to the higher gas accessibility of iron oxide on the particle surface.
4. The formation of metallic iron results in less absorption of microwaves owing to a shorter penetration depth than the case of either wüstite or magnetite. Lower microwave absorption then leads to a decrease in the sample temperature during microwave heating.
